# 

**Published:** 1889-10-12

**Authors:** 


### SATURDAY, OCTOBER 12th, 1889.

**Bumbledom at Birmingham** - 17
**Words of Consolation**—Forgiveness of Sin - 18
**A Phase of Modern Medicine** - 19
**The Doctor's Pulpit.**—A Fresh Start - 21
**The Borderland of Madness.** By the Rev. P. H. Good, M.A. 22
**Editor's Letter-Box.**—Hypnotism - 22
**Everybody's Page** - 23
**Notes and News** - 24
**Annotations.**—Smokeless London—Half Madness and Responsibility—The Barrister's Tea - 26
**Royal Hospital for Diseases of the Chest** - 31
**Scraps and Gleanings** - 32

**The Practitioner's Outlook and Retrospect:**
Medicine—Scurvy - Ainhum—Microbes - 27
Surgery—Progressive Paralysis—Fractured Patella - 27
Recent Research—Transfusion of Blood—Detection of Human Blood - 28
New Drugs, Appliances, and Things Medical—"Christia" and Modern Day Poultices - 28
The Winter Session 1889-90: Introductory Addresses
Westminster Hospital - 29
St. George's Hospital—St. Thomas's Hospital—University College Hospital—The Yorkshire College, Leeds—The Sheffield Medical School - 30

**EXTRA SUPPLEMENT—THE NURSING MIRROR.**

**En Passant.**—Bournemouth News—Village Nursing—Mary Adelaide Letter—Secession to Rome—Short Items—Nurses in Australia - vii
**Case Taking.**—Third Paper - viii
**"The Bitter Cry of 'They.'"** By One of Them - viii
**Examination Questions.**—A New Competition - ix
**Everybody's Opinion.**—Superannuated at Forty-three—Nursing in Workhouses—Asylum Attendants—Nurse or Doctor - ix
**Appointments—Vacancies—Notes and Queries** - x
**Amusements and Relaxation** - x
**A New Year's Gift** - xi

